# Supplementary material for: Low Light Increases the Abundance of Light Reaction Proteins: Proteomics Analysis of Maize (Zea mays L.) Grown at High Planting Density
Source: Int J Mol Sci. 2022 Mar 10;23(6):3015. doi: 10.3390/ijms23063015 (PMC8955883; doi:10.3390/ijms23063015)
Supplement: Supplementary file 1 [file ijms-23-03015-s001.zip › Supplementary file 2-The details of the TMT-based proteomics analysis methods.pdf]

Details of TMT-based quantitative proteomics analysis methods

Sample preparation

According to the previous studies, we chose the 0, 20 and 40 days after anthesis as three distinct phases during which to determine the leaf proteins expression changes during the stages of grain yield formation. Samples were collected after physiological measurement in each stages. The middle portions of the leaves (the veins removed) were collected and frozen in liquid nitrogen and stored at -80℃ prior to proteomics analysis.

Protein Extraction and quantitative analysis

Proteins were extracted from frozen leaves (3 plant densities × 3 growth stages) with three biological replicates. Briefly, the samples were ground into powder in liquid nitrogen and homogenized in lysis buffer (1 M Sucrose, 0.5 M Tris-HCl (pH 8.0), 0.1 M KCl, 50mM Ascorbic acid, 1% NP40, 1%NaDOC, 10mM EDTA, 10 mM dithiothreitol (DTT) and 1% protease inhibitor Cocktail). The extraction was mixed with ice-cold Tris buffer phenol (pH 8.0) and the protein supernatants were transferred to new tube after centrifugation (5500 × g, 10 min, 4 ℃), and then precipitated with ice-cold ammonium acetate/methanol at -20 ℃ overnight. Next, the sediment pellets after centrifugation at 16,000 × g for 10 min at 4 ℃ were rinsed with ice-cold methanol at -20 ℃. This process was then repeated twice with ice-cooled acetone, vacuum-dried and stored at -80 ℃. The lysis buffer (8 M Urea, 50 mM Tris-HCl (pH 8.0), 1% NP40, 1% NaDOC, 10 mM EDTA, 5 mM DTT and 1% protease inhibitor Cocktail) was added to the obtained pellets, and then collected the protein supernatants after centrifugation (20,000 × g, 15 min, 4 ℃). The protein concentration was quantified using the 2D Quant kit (GE Healthcare Bioscience, Shanghai, China), and the quality of protein extraction was detected by sodium dodecyl sulfate polyacrylamide gel electrophoresis (SDS-PAGE).

Trypsin digestion, TMT labeling and high-performance liquid chromatographer (HPLC) fractionation

A total of 10 protein samples, including an internal standard (ISTD: 50 μg of each protein samples was equally mixed), were digested with Trypsin. For trypsin digestion, the protein samples were reduced with 5 mM DTT for 30 min at 30 ℃. 30 mM iodoacetamine (IAA) was then added to alkylate the proteins for 45 min at room temperature (RT) in darkness. The proteins was precipitated with ice-cold acetone at -20 ℃ overnight. The obtained pellets after centrifugation (20,000 × g, 10 min, 4 ℃) were rinsed thrice with ice-cold acetone at -20 ℃ and dried. The trypsin was then added to the protein samples which contain with 100 mM triethylammonium bicarbonate (TEAB) at 37 ℃ for overnight digestion.

The digested peptides were labeled using TMT 10-plex Isobaric Label Reagent Set (Thermo Fisher Scientific, San Jose, CA, USA) following procedures recommended by the manufacturer (Fig. S1). Briefly, one unit of TMT reagent (defined as the amount of reagent required to label 100 g of protein) was reconstituted in 24 l acetonitrile (ACN). The

peptide was dissolved in 0.5 M TEAB, mixed with the TMT reagent and incubated for 2 h at RT. The digested peptides were desalinated by Strata-X C18 SPE column (Phenomenex, USA) and vacuum-dried.

Then, the labeled peptides were redissolved in buffer A (100% H_2O, 5 mM NH_4OH, pH 10.0), and pooled together followed by centrifugation (20,000 × g, 2 min). The supernatant was fractionated using a Waters XBridge Shield C18 RP column (3.5 μm, 2.1 × 150 mm) on a Shimadzu LC20AD high-performance liquid chromatographer (HPLC) operating at 1 mL/min under high-pH reverse-phase condition. The separation gradient as follows: 0-80 min, liner gradient from 5% increased to 80% buffer B (80% ACN, 5 mM NH_4OH, pH 10.0); 80-90 min, liner gradient from 80% down to 5% buffer B. Finally, combined the obtained 60 fractions into 20 fractions and vacuum-dried.

LC-MS/MS Analysis

The vacuum-dried samples were dissolved in buffer A (100% H_2O, 0.1% formic acid (FA), and the supernatant after centrifugated (20,000 × g, 2 min) was analyzed using an EASY-nLC UPLC system (Ultimate RSLCnano 3000, Dionex, Sunnyvale, CA, USA) connected to Q Exactive HF mass spetrometer (Thermo Fisher Scientific, San Jose, CA, USA). The sample was loaded onto a reversed-phase pre-column (Thermo Scientifi Acclaim PepMap 100 C18 column, 75 μm × 20 mm, 2 μm) using an autosampler at a flow rate of 250 nL/min and separated by a reversed-phase analytical column (Thermo Acclaim PepMap RSLC C18 column, 75 μm × 500 mm, 2 μm) with the following gradient: 0-62 min, linear gradient from 2% increased to 80% buffer B (80% ACN, 0.1% FA); 62-70 min, linear gradient from 80% down to 2% buffer B.

The mass spectrometer was operated in positive ion mode, and MS spectra were acquired over a range of 350-1800 m/z. For MS and MS/MS scans, the resolution for the orbitrap fusion was set at 60,000 and 30,000 at 100 m/z, respectively. Data-dependent acquisition mode was set as top speed, the top 20 precursor ions above a threshold ion count of 2E5 in the acquired MS scan spectrawere slected for further MS/MS analysis. The electrospray voltage and capillary tempreture were respectively set at 2.0 kV, 250 ℃. The separation window was ±0.7 m/z, and ions were fragments through higher energy collisional dissociation (HCD) with normalized collision energies (NCE) of 27%. The maximum ion injection times (maxIT) were respectively set at 50 and 100 ms for the MS and MS/MS scans. Besides, the automatic gain control (AGC) target values for master scan modes and MS/MS were set to 3E6 and 5E4, respectively. The dynamic exclusion duration was10 s.

Data analysis and Bioinformatics analysis

The resulting raw LC-MS/MS spectra were analyzed using MaxQuant search engine (version 1.4.1.2) searching against the Uniprot database. Search parameters as follows: monoisotopic mass; trypsin as cleavage enzyme; two max missed cleavages; TMT 10-plex (N-term), TMT 10-plex (K), and carbamidomethylation of cysteine as fixed modifications; and oxidation of methionine as variable modifications. Mass error was set to 10 ppm for precursor

ions and 0.02 Da for fragment ions. The false discovery rate (FDR) was set to 1% for peptide and protein identification. For database search, the length of the shortest peptidewas set to seven amino acid residues. All of the other parameters used in MaxQuant were default settings.

The TMT reporter ion intensity was used for protein quantification. At least one unique peptide was required per protein from all three replicates were used in the follow up quantification analysis. According the summed intensity of the matched spectra to quantified the protein ratios in each replicate. An arithmetic mean value of ratios of different TMT reporters in three biological replicates was used as the quantitative result of each treatment. Proteins with a p-values <0.05 by Student t-test and a fold-change of >1.30 or <0.77 were considered as Differentially expressed proteins (DEPs). The gene ontology (GO) database (http://geneontology.org/) was used to determine all the differentially expressed protein categories (DEPs). The Kyoto Encyclopedia of Genes and Genomes (KEGG) database (https://www.kegg.jp/kegg/pathway.html) was used to annotate protein pathway. GO and KEGG enrichment analyses were performed using the Fisher's exact test, and FDR correction for multiple testing was also performed.
