# Supplementary material for: Low Light Increases the Abundance of Light Reaction Proteins: Proteomics Analysis of Maize (Zea mays L.) Grown at High Planting Density
Source: Int J Mol Sci. 2022 Mar 10;23(6):3015. doi: 10.3390/ijms23063015 (PMC8955883; doi:10.3390/ijms23063015)
Supplement: Supplementary file 1 [file ijms-23-03015-s001.zip › Supplementary file 3-Figure S1+S2.pdf]

Low light increases the abundance of light reaction proteins: proteomics analysis of maize (*Zea mays* L.) grown at high planting density

Bin Zheng[#], Wei Zhao[#], Ting-hu Ren, Xing-hui Zhang, Tang-yuan Ning, Peng Liu, Geng Li[*]

College of Agronomy, Shandong Agricultural University, Tai'an, Shandong 271018, P. R. China

A. TMT Labeling Procedure

Samples

0 d	LD	ND	HD
20 d	LD	ND	HD
40 d	LD	ND	HD

Protein extraction and trypsin digestion

↓

TMT Labeling

Sample groups		Labeling information
	LD	126
0 d	ND	127 N
	HD	127 C
	LD	128 N
20 d	ND	128 C
	HD	129 N
	LD	129 C
40 d	ND	130 N
	HD	130 C
ISTD		131

Note: three repetitions for each sample group;
ISTD: internal standard

↓

HPLC Fractionation

↓

LC-MS/MS Analysis

↓

Database Search

↓

Bioinformation Analysis

B. TMT 10plex Reagents (TMT[10])

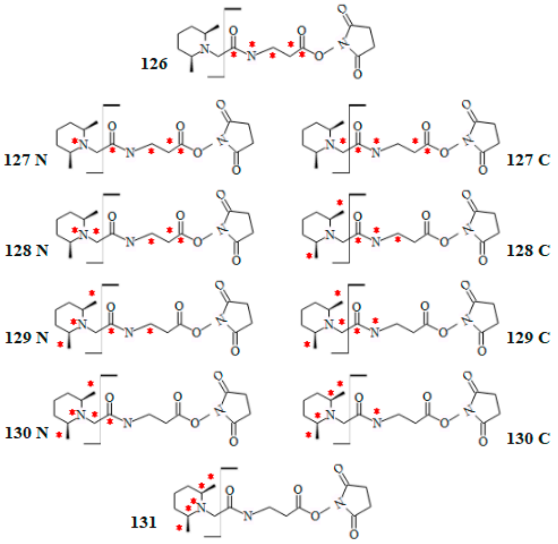

C. Mass Spectrometry Quantification

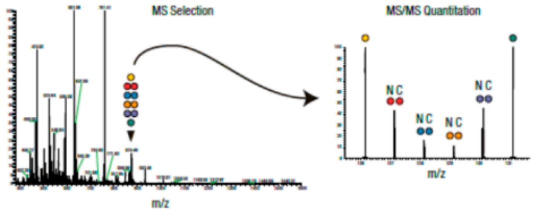

D. MS/MS Spectrum Database Search Analysis Summary

	Total Spectrums	Matched Spectrums	Peptides	Unique peptides	Identified Proteins	Quantifiable Proteins
Rep. 1	622876	103590	42881	33904	7683	7017
Rep. 2	568952	92848	41104	32646	7550	6945
Rep. 3	541887	89539	40786	32644	7470	6871
Total	1733715	285977	124771	99194	10031	9226

Supplementary Figure S1. Procedure summary for mass spectrometry (MS) experiments with TMT isobaric mass tagging reagents. (A) TMT labeling procedure. (B) TMT 10-plex reagents. (C) Mass spectrometry quantification. (D) MS/MS spectrum database search analysis summary. LD, low density; ND, normal density; HD, high density. 0, 20 and 40 d, days after anthesis.

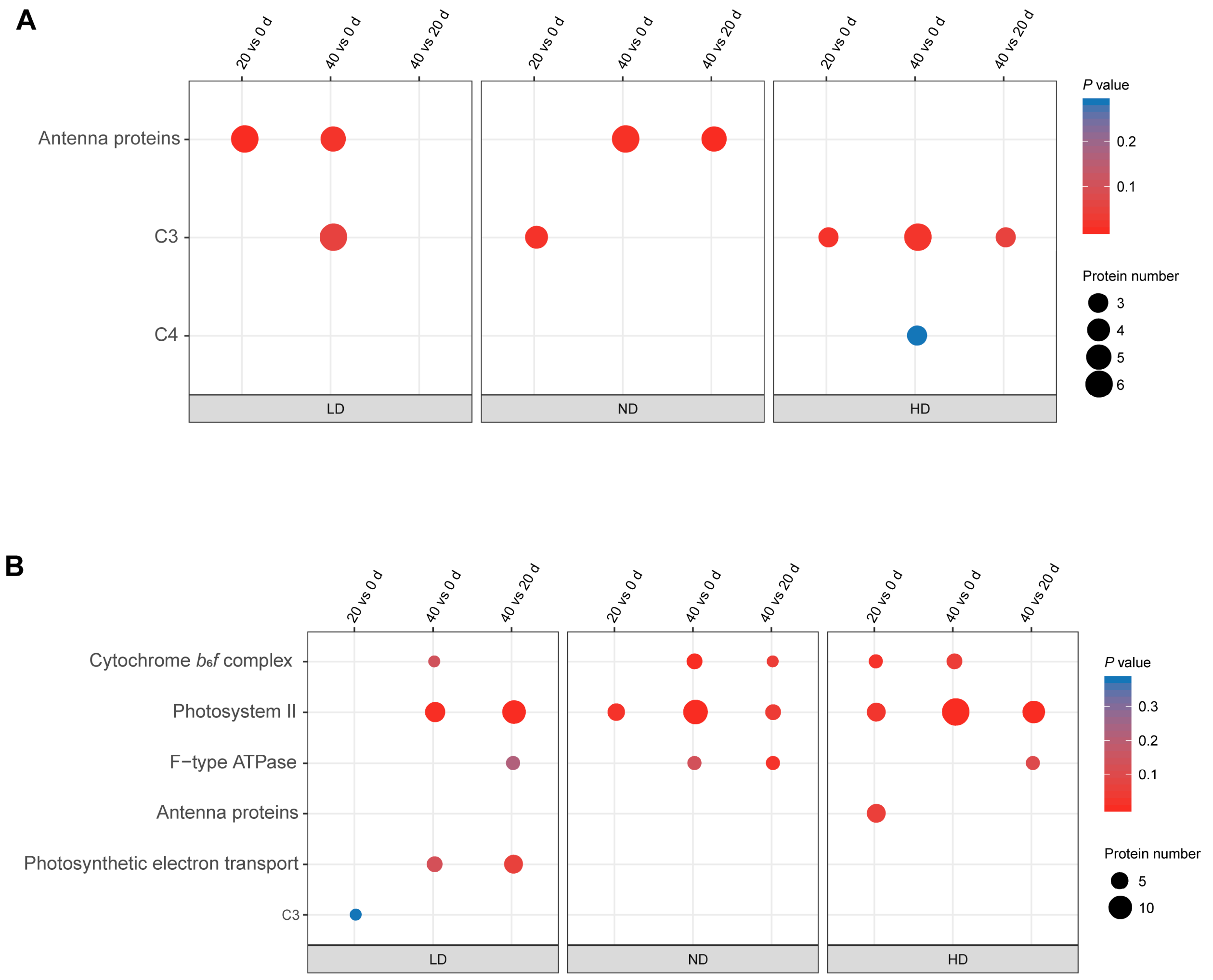

Supplementary Figure S2. Kyoto Encyclopedia of Genes and Genomes (KEGG) pathway enrichment of differentially abundant proteins (DAPs) in leaves under low (LD), normal (ND), and high density (HD) planting at a pairwise comparisons between time-points. Colored circles indicate *Z* scores (-$\log_{10}$ *P*-value). LD, low density; ND, normal density; HD, high density. 0, 20 and 40 d, days after anthesis.
